# Battling complexity with complexity: why multi-modal artificial intelligence may be the key to atrial fibrillation prediction

**DOI:** 10.1093/ehjdh/ztaf139

**Published:** 2025-12-03

**Authors:** Emma Svennberg, Ramesh Nadarajah

**Affiliations:** Karolinska Institutet, Karolinska University Hospital Huddinge, SE-141 86 Stockholm, Sweden; Leeds Institute for Data Analytics, University of Leeds, Leeds LS2 9JT, UK; Leeds Institute of Cardiovascular and Metabolic Medicine, University of Leeds, Leeds LS2 9JT, UK; Department of Cardiology, Leeds Teaching Hospitals NHS Trust, LS1 3EXLeeds, UK

Atrial fibrillation (AF) is the most common sustained arrhythmia worldwide, yet its intermittent and often silent nature means it can remain undiagnosed for long periods.^1^ Untreated AF exposes patients to the risk of serious complications, prompting initiatives aimed at earlier and more systematic detection.^2^ Traditional risk-prediction models such as the CHARGE-AF score, estimate long-term incident AF risk based on clinical characteristics and are useful for multi-year stratification but more limited for guiding immediate interventions.^3^ In contrast, more recent artificial intelligence (AI)-based approaches have emerged that seek to predict AF development using a shorter time horizon.^4^ Although AI offers the promise of integrating multiple data modalities, most current models still rely on a single modality, commonly ECG, blood biomarkers or clinical risk factors, highlighting both the opportunity, and the challenge of capturing the full complexity of AF.^4–6^

AF is increasingly recognized as a heterogeneous disease, with diverse clinical and pre-clinical presentations and a complication risk associated not only with the patients’ clinical characteristics, but also by the stage and burden of the arrhythmia.^7^ This complexity underscores the limitations of traditional, single-modality prediction tools and highlights the need for multi-modal AI, that can capture the diverse clinical trajectories of AF.

In the current study by Tarabanis *et al.*, the authors used a large dataset using 157 192 cardiologist-validated sinus rhythm 12-lead ECGs and health care data from 76 986 patients to develop five AI-based models to detect recent AF paroxysms. (REF current paper) Notably, the sinus rhythm ECGs were obtained up to 90 days before or after an ECG showing AF, reflecting the authors’ intention to mirror real-world clinical scenarios in which patients may already have had unrecognized paroxysmal AF at the time of their initial assessment. ECG data were then combined with clinical features including past medical history, demographics, vitals, and the CHARGE-AF risk score.^3^ All of the AI-developed models exceeded the performance of the traditional CHARGE-AF score, with each layer of added information providing incremental gains in discrimination. The most comprehensive model, combining sinus rhythm ECGs with all CHARGE-AF features, achieved the highest accuracy (AUC 0.89, 95% CI 0.88–0.89; AUPRC 0.69, 95% CI 0.67–0.70) in the internal test set. Importantly, this performance was preserved across external validations, including a U.S. outpatient cohort (AUC 0.90 and AUPRC 0.67), Greek tertiary hospitals (AUC 0.85 and AUPRC 0.78), and across age, sex, and race strata.

The addition of individual CHARGE-AF components produced stepwise improvements in discrimination. Incorporating past medical history variables (heart failure, myocardial infarction, and diabetes) yielded the largest incremental gain in prediction, whereas adding demographic (age, race, and smoking) and vital-sign elements (height, weight, and blood pressure) contributed more modestly.

This study illustrates that neither ECG features nor clinical risk factors alone fully capture AF risk; it is their combination that yields a markedly stronger and more reliable model. For AF prediction, the 12-lead ECG thus emerges not merely as a diagnostic snapshot but as a potential continuous risk biomarker when interpreted within clinical context. Looking ahead, incorporating dynamic or longitudinal changes in both ECG and clinical variables may offer an even more powerful way to track and refine individual AF risk over time to deepen our prediction of if, and when, AF may develop. Such an approach could enable truly targeted screening interventions, personalized not only to who should be screened but also to the optimal timing of that screening.

Ultimately, though, prediction alone is not the goal. The value of multi-modal AI lies in whether earlier and more accurate identification of at-risk individuals can be used to guide interventions that prevent adverse outcomes for our patients. Most modelling studies are conducted in retrospective data, which limits our understanding of how they can improve patient care and outcome. Prospective evaluation is essential to determine their real-world utility and the window of optimal prediction that will be truly actionable for clinical decision-making. Future studies therefore need to move beyond demonstrating ever-higher AUCs to showing that AI-guided screening and management strategies improve patient-centred outcomes and are cost-effective at scale.
